# Comparative Analysis of Ankle Muscle Activation During Sitting and Standing in Individuals with Neck Pain: An Electromyographic Study

**DOI:** 10.3390/jcm15218755

**Published:** 2026-11-09

**Authors:** Saad Z. Alqahtani, Marwan M. A. Aljohani, Raghad S. Alamri, Rahaf K. Albeladi, Aseel A. Alsamiri, Jana A. Alahmadi, Bayader N. Al-Harbi, Alaa M. Alqurafi, Ahmed S. Alanazi, Walaa M. Ragab

**Affiliations:** Department of Physical Therapy, College of Medical Rehabilitation Sciences, Taibah University, Madinah 42241, Saudi Arabia; mmjohani@taibahu.edu.sa (M.M.A.A.); 4455399@taibahu.edu.sa (R.S.A.); 4456550@taibahu.edu.sa (R.K.A.); 4455071@taibahu.edu.sa (A.A.A.); 4350183@taibahu.edu.sa (J.A.A.); 4351631@taibahu.edu.sa (B.N.A.-H.); aqurafi@taibahu.edu.sa (A.M.A.); asanazi@taibahu.edu.sa (A.S.A.); wragab@taibahu.edu.sa (W.M.R.)

**Keywords:** neck pain, Numerical Rating Scale, Neck Disability Index, craniovertebral angle, surface electromyography

## Abstract

**Background/Objectives:** This study compared upper trapezius (UT), peroneus longus (PL), medial gastrocnemius (MGC), and tibialis anterior (TA) activation between university students with and without neck pain during sitting, standing, and writing. **Methods:** This cross-sectional study included 27 students: 19 with neck pain and 8 controls. Pain intensity and disability were assessed using the Numerical Rating Scale and Neck Disability Index. Bilateral surface electromyographic root-mean-square amplitudes were normalized to maximal voluntary contractions. Linear mixed-effects models assessed muscle activity, with Bonferroni-adjusted pairwise comparisons. Within-group positional analyses were exploratory. **Results**: Overall UT amplitude was higher in the neck pain group (mean difference: 6.117; 95% confidence interval: 3.045–9.190; *p* < 0.001). Within this group, UT amplitude was higher during sitting (mean difference: 5.937; *p* = 0.006) and writing than standing (mean difference: 9.212; *p* < 0.001), and higher on the right than the left (*p* = 0.002). PL and MGC amplitudes were higher during standing than sitting in both groups. Within the neck pain group, both muscles also showed higher amplitudes during writing than sitting. TA amplitude was higher during standing than during sitting (*p* = 0.002) and writing (*p* = 0.035) within the neck pain group. **Conclusions:** Neck pain was associated with higher overall UT activity. Positional differences in ankle muscle activity in patients with neck pain were also observed; however, additional studies with larger samples will be required to explore this observation further.

## 1. Introduction

Neck pain (NP) is a common burden affecting the general population; it peaks in middle age and is more prevalent in women [1]. According to the 2021 Global Burden of Disease study, an estimated 203 million people worldwide had neck pain in 2020, with an projected increase to 269 million by 2050 [2]. A study of Saudi university students found that 59.1% of participants reported neck or shoulder pain while using computers or smartphones [3]. Neck pain is defined as pain experienced in the cervical region, with or without radiation to one or both upper limbs, lasting for a minimum of one day [4]. Pain lasting more than three months is considered chronic pain [5]. Forward head posture (FHP) is becoming increasingly common with the rise of technology and smartphones among younger generations, and it is associated with neck disorders [6]. FHP is a result of postural deviation that is associated with increased activity and subjective fatigue of superficial muscles such as the upper trapezius (UT); hence, long-term neck displacement is suggested as a risk factor for neck pain [7].

Postural stability involves the activation of lower limb muscles [8]. Previous studies have reported that postural stability is affected in patients with chronic neck pain, such as those with whiplash-associated disorders [9,10]. Another study found increased postural instability and neck muscle activities during different balance tasks in patients with whiplash-associated disorders [11]. These findings suggest that neck pain may be associated with changes in muscle recruitment beyond the cervical region during postural tasks. Based on that, examining UT and ankle muscle activity across sitting, standing, and writing may help determine whether activation patterns differ according to neck pain status and task demands.

The trapezius muscle stabilizes the scapula and contributes to movement of the head and neck; however, changes in its stiffness have implications for cervical mobility in asymptomatic young people [12,13]. The majority of studies in the literature related to neck pain mainly investigate the neck and shoulder muscles. For instance, a previous study found that UT muscle activation was significantly higher during box transfers to ankle- and shoulder-height shelves than during transfers to knee- and waist-height shelves [14]. Another study found that pain and muscle fatigue manifested in median frequency of neck muscle activity, where UT activities differed based on neck flexion angles while using smartphones for ten minutes [15]. These findings suggest that neck muscle activities change during different positions.

Although a previous study has examined UT activity and lower-limb muscle activity during standing balance tasks [16], it remains unclear whether UT and ankle muscle activation patterns differ between university students with and without neck pain across sitting, standing, and writing tasks. Therefore, the principal aim of this study was to compare overall UT activity between university students with and without neck pain across standing, sitting, and writing tasks using surface electromyography (sEMG). Exploratory analyses examined ankle muscle activity, positional and side differences, and associations among UT activity, ankle muscle activity, and craniovertebral angle.

## 2. Materials and Methods

### 2.1. Sample and Subjects

**Figure 1:**
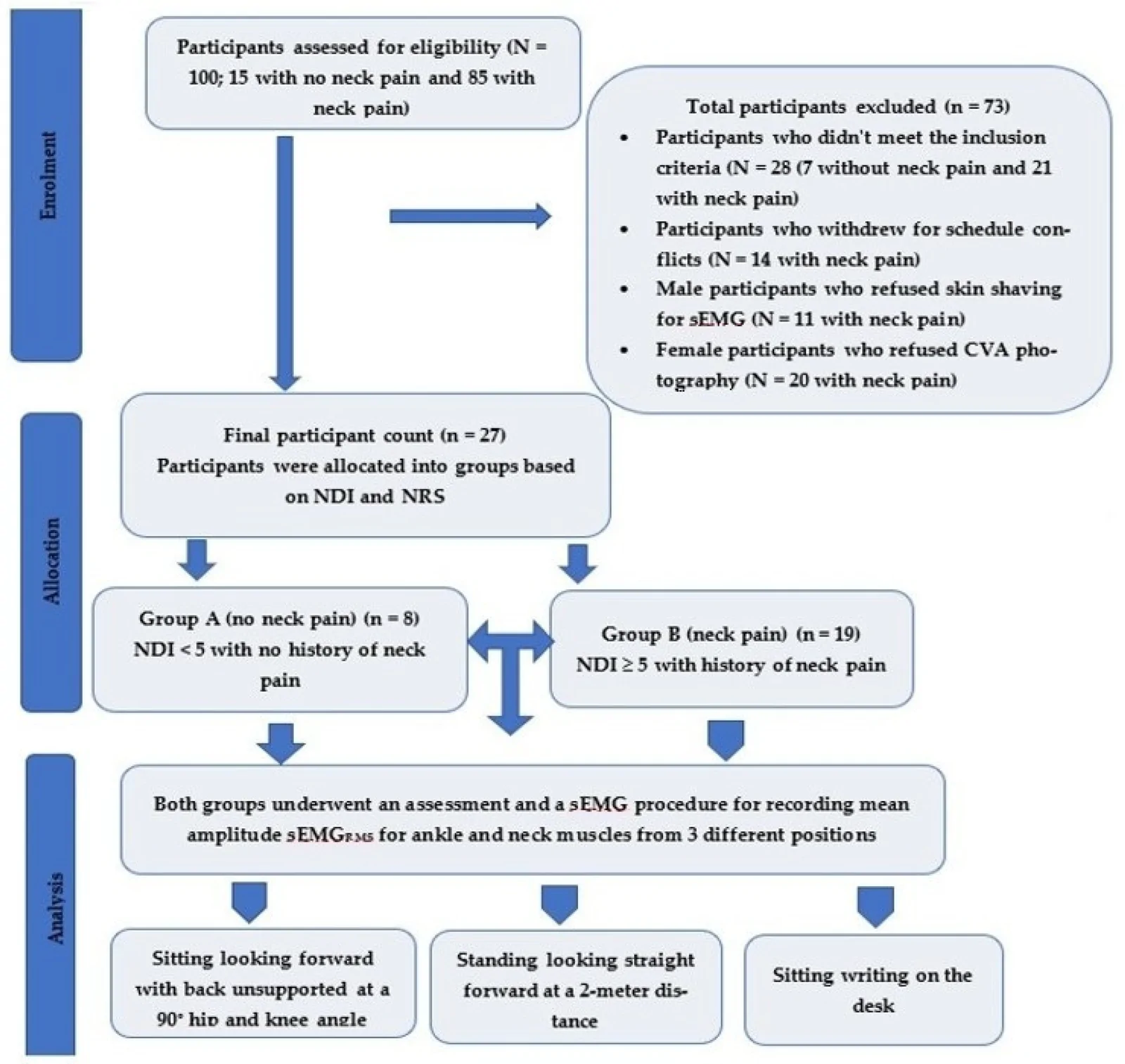
Flowchart illustrating the recruitment and grouping of participants.

This is a cross-sectional case–control study that was conducted at the College of Medical Rehabilitation Science (CMRS) at Taibah University, Saudi Arabia. A convenience sample of 27 university students were recruited for the study and assigned into two groups: Group A included individuals with no neck pain (*n* = 8), and Group B included individuals with neck pain (*n* = 19) (Figure 1). The participants were recruited from the CMRS through classroom announcements made with instructors’ permission. The researchers delivered a one-minute neutral script outlining the study’s purpose, participation requirements, and the voluntary nature of participation. Additional recruitment was conducted via WhatsApp messenger (Meta Platforms, Melano Park, CA, USA; Version 2.24.25) by distributing an announcement about a study among CMRS students.

Purposive sampling was employed according to predefined eligibility criteria. Randomization was not applicable because group allocation was determined by the participants’ neck pain status rather than by experimental assignment. The postural conditions—including sitting, standing, and writing tasks—represented a within-subject factor and were assessed across all participants. The study protocol was approved by the Institutional Review Board of the CMRS-REC Committee at Taibah University (Approval No: TU-RHB-26-02-006). Written informed consent was obtained from all participants prior to their participation.

### 2.2. Sample Size

Sample size calculation was performed using G*Power 3.1 software based on a pilot study involving 4 students (2 in each group). The primary outcome was the mean amplitude of Surface Electromyography Root Mean Square (sEMGRMS) measured by Noraxon from the sitting, standing, and writing tasks. Based on an effect size of 0.95, alpha = 0.05, and power = 80%, the required sample size was estimated at 22 participants (11 per group).

### 2.3. Eligibility Criteria

Participants were included in the study if they were university students aged between 18 and 22 years old with a body mass index (BMI) ranging from 18.5 to 24.9 kg/m^2^. Neck-related disability was assessed using the Neck Disability Index (NDI). Participants in the neck pain group were recruited if they reported a neck pain intensity of ≥2/10 on a Numerical Rating Scale (NRS) for more than three months and had an NDI score of ≥5, corresponding to mild (5–14), moderate (15–24), severe (25–34), or complete disability (35–50). All participants in the neck pain group reported centralized neck pain and had to be able to understand and independently follow the test instructions and demonstrate sufficient postural control to maintain unsupported standing for at least two minutes. Eligibility for the control group required the absence of persistent neck pain for a minimum of three months prior to participation, in addition to having an NDI score below 5.

Participants were excluded if they had any neurological disorders such as epilepsy, vestibular diseases, lower-limb injuries, diagnosed cervical disc herniation, cervical spondylosis, whiplash-associated disorder, inflammatory arthropathy, myelopathy, radiculopathy, congenital cervical spine anomalies, or malignancy. Individuals with diabetes mellitus accompanied by neuropathy, those using medications known to affect neuromuscular function, those with neurological disorders, and those who were pregnant were also excluded from the study.

### 2.4. Assessment Tools and Procedure

A detailed clinical history was obtained from each participant, including any history of vertigo with neck motion or ear pain. Current neck pain intensity was assessed using the NRS, along with supplementary information regarding pain characteristics, onset, and duration, to confirm the presence of chronic neck pain. The NRS was used as a screening tool to quantify current pain intensity, requiring participants to rate their pain on a scale from 0 (no pain) to 10 (worst imaginable pain) [17].

The effect of neck pain on activities of daily living (ADL) was also assessed using the NDI. This scale is intended to evaluate self-reported neck-related impairments and determine the impact of neck issues on everyday activities, including personal care, employment, and leisure pursuits. The NDI is a 10-item self-reported questionnaire that measures the level of dysfunction. Participants who reported neck pain through the NRS were then asked to answer 10 questions (1 per item) by the NDI, each with 6 possible responses scored from 0 to 5, with 5 indicating the most severe disability. Then, the total raw score was divided by the total possible score and multiplied by 100 to give a percentage of disability [18].

FHP was also assessed using the craniovertebral angle (CVA); a CVA of less than 50° was considered to be indicative of forward head posture (FHP). The angle is defined as the intersection between a horizontal line passing through the spinous process of the seventh cervical vertebra (C7) and a line connecting C7 to the tragus. A smaller CVA indicates more pronounced FHP [19].

In this study, a smartphone application (PhysioMaster; physical therapy goniometer and posture analysis, version v2.5.7) was used to measure the CVA, and all images were captured using an iPhone 14 positioned at a fixed distance and height to ensure consistency. This application is considered to be a valid and reliable tool to measure the CVA. This method has several advantages: it is reasonably quick, the images and values are obtained easily, and it is more accurate and reliable than visual evaluation alone. Following a standardized protocol, all images were captured in the sagittal plane under two conditions: First, the participants were instructed to stand in an erect posture while looking straight ahead, with their arms relaxed at their sides. A lateral photograph was then taken from a distance of 0.5 m at the shoulder level. Second, the participants were asked to sit in an upright position without back support, and a second image was captured under the same conditions, as described previously [20]. Anatomical landmarks were identified by the researcher through palpation of the C7 spinous process and marked using adhesive stickers. The tragus served as the second reference point and was also marked using adhesive stickers. The CVA was determined by drawing a horizontal line through C7 and a second line extending to the tragus, after which the angle was automatically computed by the application. All landmark palpations, markings, image acquisitions, and angle measurements were performed by the same examiner to ensure consistency throughout data collection.

Muscle activation patterns were quantified using a wireless surface electromyography (sEMG) system integrated with an internal inertial measurement unit (Ultium EMG; Noraxon USA Inc., Scottsdale, AZ, USA); the Noraxon Myoresearch (MR3) system, version 12. Bioelectrical signals were captured using Noraxon HEX Dual disposable, self-adhesive Ag/AgCl snap electrodes. A fixed inter-electrode spacing of 2.0 cm was used to ensure consistency in signal recording and minimize crosstalk.

A participant profile was created for every participant using the Noraxon MR3 software by entering the required demographic information into the database. The EMG sensors were then connected and verified through the software interface to ensure accurate signal detection and synchronization. Subsequently, the appropriate measurement module and channel configuration were selected based on the muscle evaluated.

The participants were asked to refrain from any vigorous physical activity for at least 24 h before the experiment. The skin under each electrode site was prepared by removing hair when necessary and cleaning the area with isopropyl alcohol to reduce skin impedance and improve signal quality. Surface EMG electrodes were placed according to the SENIAM recommendations [21]. For the UT, the electrodes were positioned midway between the C7 spinous process and the acromion at the most prominent point identified during shoulder elevation. For the TA, PL, and MGC, the electrodes were placed over the most prominent muscle belly during dorsiflexion, eversion, and plantarflexion, respectively. Specifically, the TA electrode was positioned at one-third of the distance between the fibular head and the medial malleolus according to SENIAM.

The sEMG electrodes were placed bilaterally over four muscles—the upper trapezius (UT), medial gastrocnemius (MGC), tibialis anterior (TA), and peroneus longus (PL)—following standardized anatomical landmarks. A total of eight muscle-specific electrodes were applied (four per side).

Following electrode placement over the targeted muscles, the participants performed maximum voluntary isometric contractions (MVCs) against manual isometric resistance for each muscle from the sitting position. Each MVC was performed for 3–5 s, with three trials collected for each muscle and a 5 s rest period provided between trials to minimize fatigue. The highest MVC value (in microvolts) obtained across the trials was used as the reference value for EMG normalization. Subsequently, the participants performed the experimental tasks under three conditions: First, they were seated on a chair with their hips and knees flexed to 90° while looking straight ahead at a target positioned 2 m away. Second, they performed an upright standing position while maintaining the same visual focus. To ensure consistency across participants, the visual target was positioned at each participant’s eye level using a laser pointer and an adhesive marker placed on the wall at a distance of 2 m. The participants were instructed to maintain their gaze on the target throughout each testing condition—sitting and standing. The purpose of this procedure was to promote a neutral forward gaze and minimize unnecessary variations in head orientation during data collection. Verbal instructions were provided, and the participants were monitored throughout testing to ensure compliance with the task requirements. Third, the participants completed a writing task in a self-selected comfortable posture. To minimize variability across participants for the writing task, the sitting position was standardized. The chair height was adjusted individually to achieve approximately 90° of knee flexion with the feet supported on the floor. In addition, all participants performed the same writing task using a tablet placed on the desk throughout data collection.

The sequence of postural tasks was not fixed across participants: some participants began with the sitting condition, whereas others began with the standing condition. Rest periods were provided between tasks as needed, in order to avoid fatigue.

During each condition, sEMG recordings were initiated using the MR3 software, and muscle activity was recorded in real time for 3–5 s. Trials of MVCs were used to normalize EMG data, providing a standardized reference for comparison across participants and conditions. EMG signals obtained during sitting, standing, and writing tasks were then amplitude-normalized to the corresponding MVC, smoothed, and processed to calculate the mean amplitude sEMGRMS value for each muscle.

### 2.5. Statistical Analysis

Data were analyzed using IBM SPSS Statistics (version 31.0.2.0 IBM Corp., Armonk, NY, USA). The normality of data distribution and homogeneity of variance were assessed using the Shapiro–Wilk and Levene tests, respectively. Since age, weight, height, BMI, and CVA were non-normally distributed and the groups had unequal sample sizes, the Mann–Whitney U test was used to compare between Groups A and B. Due to the unequal sample size, a linear mixed-effects model was employed to examine the effects of posture (sitting, standing, and writing tasks) and group (neck pain vs. no neck pain) on the activation of the UT, TA, PL, and MGC muscles. Posture was treated as a within-subject factor, whereas group was treated as a between-subject factor. Furthermore, Spearman’s correlation coefficient was used to correlate the mean amplitude sEMGRMS of the UT with ankle muscles and CVA with muscle activity from different positions; statistical significance was set at *p* < 0.05.

## 3. Results

A total of 100 students were screened for eligibility; of these, 28 did not meet the inclusion criteria, 14 withdrew due to scheduling conflicts with lecture times/exams or because of transportation schedules, 11 male students refused to have their skin shaved for applying EMG, and 20 female students refused to have their pictures taken to measure their CVA. Therefore, 27 students completed the study and were assessed for ankle and neck muscle activity in standing, sitting, and writing tasks for both groups. Based on NRS and NDI assessment, the participants were classified into two groups: those who scored less than 5 in addition to having no history of neck pain were assigned to Group A (GA, *n* = 8), while those who scored more than or equal to 5 on the NDI, in addition to having a history of neck pain, were assigned to Group B (GB, *n* = 19). The demographic characteristics of the two groups are presented in Table 1 and Table 2.

Statistical analysis using the Mann–Whitney U test revealed no significant differences between groups in terms of age (*p* = 0.053), weight (*p* = 0.150), height (*p* = 0.082), body mass index (BMI) (*p* = 0.385), CVA from sitting position (*p* = 0.669), or CVA from standing position (*p* = 0.163), indicating baseline comparability. These findings confirm demographic homogeneity across all groups.

### 3.1. Analysis of Mean Amplitude sEMGRMS for UT Muscle Between and Within Groups

The results of the between-group comparison showed a significant difference in the activation of the UT (F = 15.595, *p* < 0.001), with more significant activation in Group B than in Group A, while no significant difference was observed between groups in the side comparison (F = 0.806, *p* = 0.371). Moreover, there were significant differences between groups regarding the three different positions (F = 4.806, *p* = 0.010). UT pairwise comparisons with Bonferroni adjustment revealed that the mean amplitude sEMGRMS of the UT from a standing position was significantly less than for the writing task (mean difference = −5.530, *p* = 0.013) (Table 3).

The results of the within-group comparison showed no significant difference in GA regarding side comparison (F = 0.889, *p* = 0.358) or positions changing (F = 0.597, *p* = 0.561), while GB showed significant differences in the activation of the UT with changing positions (F = 12.815, *p* < 0.001). UT pairwise comparisons with Bonferroni adjustment revealed that the mean amplitude sEMGRMS of the UT from a standing position was significantly less than for the writing task (mean difference = −9.212, *p* < 0.001), and more significant activation was observed from a sitting compared to a standing position (mean difference = 5.937, *p* = 0.006). Furthermore, there were significant differences in the side comparison in GB, with a more significant mean amplitude sEMGRMS activation of the right side compared to the left (mean difference = 4.932, *p* = 0.002) (Table 4).

### 3.2. Analysis of Mean Amplitude sEMGRMS for PL Muscle Between and Within Groups

The results of the between-group comparison showed no significant difference between the two groups in the side comparison of mean amplitude sEMGRMS activation for the PL (F = 0.462, *p* = 0.498) while there was a significant difference in mean amplitude sEMGRMS activation for the PL between different changing positions (F = 5.514, *p* = 0.005). PL pairwise comparisons with Bonferroni adjustment revealed that the mean amplitude sEMGRMS for the PL from a standing position was significantly higher than from a sitting position (mean difference = 6.541, *p* = 0.004) (Table 5).

Between GA and GB, no significant differences were observed in the side comparison (F = 10.011, *p* = 0.987 and F = 1.850, *p* = 0.177, respectively). However, within GB, there were significant differences in the activation of the PL with changing positions (F = 4.917, *p* = 0.010). PL pairwise comparisons with Bonferroni adjustment revealed that the mean amplitude sEMGRMS for the PL in the writing task was significantly higher than from a sitting position (mean difference = 4.830, *p* = 0.045), and from a standing position compared to a sitting position (mean difference = 5.655, *p* = 0.014). Furthermore, GA showed significant differences in the activation of the PL with changing positions (F = 10.011, *p* = 0.001). PL pairwise comparisons with Bonferroni adjustment revealed that the mean amplitude sEMGRMS of the PL from a standing position was significantly higher than from a sitting position (mean difference = 7.427, *p* = 0.002) and for the writing task (mean difference = 6.334, *p* = 0.007) (Table 6).

### 3.3. Analysis of Mean Amplitude sEMGRMS for MGC Muscle Between and Within Groups

The between-group comparison showed no significant difference between groups in the side comparison (F = 0.333, *p* = 0.565), while there were significant differences between groups with changes in positions (F = 8.075, *p* < 0.001). MGC pairwise comparisons with Bonferroni adjustment revealed that the mean amplitude sEMGRMS of the MGC from a standing position was significantly higher than from a sitting position (mean difference = 3.392, *p* < 0.001) and during the writing task (mean difference = 2.370, *p* = 0.022) (Table 7).

The within-group comparison showed no significant side differences within GA or GB (F = 0.096, *p* = 0.762 and F = 1.093, *p* = 0.299, respectively), while within GB, there were significant differences in the activation of the MGC with changing positions (F = 9.299, *p* < 0.001). MGC pairwise comparisons with Bonferroni adjustment revealed that the mean amplitude sEMGRMS for the MGC during the writing task was significantly higher than from a sitting position (mean difference = 2.013, *p* = 0.025), and from a standing position, it was significantly higher than from a sitting position (mean difference = 3.18, *p* < 0.001) (Table 8).

For Group A, there were also significant differences between positions (F = 17.010, *p* < 0.001). MGC pairwise comparisons with Bonferroni adjustment revealed that the mean amplitude sEMGRMS for the MGC from a standing position was significantly higher than from a sitting position (mean difference = 3.602, *p* < 0.001) and for the writing task (mean difference = 3.572, *p* < 0.001) (Table 8).

### 3.4. Analysis of Mean Amplitude sEMGRMS for TA Muscle Between and Within Groups

The between-group comparison showed significant differences between the groups in terms of side comparison or position changes (F = 0.139, *p* = 0.710 and F = 2.078, *p* = 0.130, respectively).

The within-group comparison for the TA showed no significant differences within GA regarding side comparison or position changes (F = 2.337, *p* = 0.144 and F = 2.955, *p* = 0.078, respectively). Within GB, there were no significant differences regarding side comparison (F = 0.289, *p* = 0.593), while there were significance differences with changes in position (F = 6.857, *p* = 0.002). TA pairwise comparisons with Bonferroni adjustment revealed that the mean amplitude sEMGRMS for the TA from a standing position was significantly higher than from a sitting position (mean difference = 2.905, *p* = 0.002) or during the writing task (mean difference = 2.087, *p* = 0.035) (Table 9).

### 3.5. Correlation Between UT and Ankle Muscle Activation in Patients with and Without Neck Pain

In GB, UT activity in a sitting position showed significant moderate positive correlations with the PL (*p* = 0.003), suggesting coordinated activation patterns. From a standing position, UT activity also showed significant moderate positive correlations with the PL and TA (*p* < 0.001, *p* = 0.002, respectively). Meanwhile, during the writing task, UT activity showed significant moderate positive correlations with the MGC (*p* = 0.004). In GA, from a standing position, the UT showed a high-to-very-high negative correlation with the MGC (*p* = 0.005), and during the writing task, it was correlated with the PL (*p* < 0.001), MGC (*p* = 0.005), and TA (*p* = 0.016) (Table 10).

### 3.6. Correlations of CVA with Mean Amplitude sEMGRMS of UT and Ankle Muscles

In GB, from a standing position, a strong negative correlation was observed between CVA and PL activity only (*p* = 0.050), indicating that smaller CVA (more forward head posture) was associated with higher PL muscle activity. No statistically significant correlations were found between CVA and other muscles from either the sitting or standing position, although there was a wide range of correlations between them (Table 11).

## 4. Discussion

The final group allocation deviated from the intended allocation because the number of eligible students without neck pain was limited. Since neck pain is common among university students, recruiting a large pain-free control group was challenging—a finding that is supported by the study by Elsiddig et al. (2022) [3].

The present study found a higher overall sEMGRMS amplitude for UT in the neck pain group compared to the control group. A broadly similar finding was reported by Leonard et al. (2010), who found higher UT activity in symptomatic than in asymptomatic students during writing [22]. However, their writing task lasted 30 min, whereas the EMG recording in our study lasted 3–5 s. Within the neck pain group, UT muscle activity was significantly higher during sitting and writing than during standing. A previous study also reported differences in UT activity across different typing positions in individuals with chronic neck pain [23]; however, that study examined typing postures rather than sitting and standing.

Within the neck pain group, our study also found asymmetric activation of the UT, with greater activity on the right than the left. Moreover, no significant difference in the mean amplitude sEMGRMS of UT activation was detected between sides in participants without neck pain. In the present study, the PL and MGC sEMGRMS amplitudes were higher during standing than sitting in both groups. This increased activation may relate to their roles in postural control and maintaining standing balance [24]. Since this finding was observed in both groups, it might be related to the demands of standing rather than changes due to neck pain.

Within the neck pain group, the PL and MGC sEMGRMS amplitudes were significantly higher during the writing task than during sitting. TA activation was significantly higher during standing than during sitting and writing within the neck pain group. Exploratory analyses within the neck pain group showed that UT activity was positively correlated with PL and TA activity during standing. A negative correlation between the craniovertebral angle and PL activation was observed during standing. Furthermore, our study found a significant positive correlation between UT and MGC activity during the writing task. A negative correlation was observed between UT and MGC in control participants during standing. These associations should be interpreted cautiously, because multiple correlations were examined—particularly the CVA-PL association (*p* = 0.050). These associations do not establish shared neural control or compensatory mechanisms.

Several limitations should be acknowledged. Some males declined participation because they were unwilling to shave their hair at the electrode sites. Additionally, some female participants refused to be photographed for CVA assessment. These recruitment barriers may have introduced selection bias and limited generalizability. The a priori sample size estimation was based on an effect size derived from a very small pilot sample (four participants), which may have overestimated the true effect. Furthermore, unequal group allocation due to recruitment constraints may have reduced the power of some between-group comparisons despite the use of linear mixed-effects modeling. Therefore, nonsignificant between-group findings should not be interpreted as evidence of equivalence. Further studies would be needed to compare muscle activation during various habitual writing positions, as the participants in our study were allowed to adopt a comfortable writing posture during the task, which may have resulted in some residual variability in their trunk and head positions. Our intention was to evaluate muscle activity during participants’ habitual writing behaviors rather than under a strictly controlled laboratory posture, and we acknowledge this as a limitation of the present study.

## 5. Conclusions

Based on the findings of this study, overall upper trapezius activity was higher in participants with neck pain compared to those without neck pain. The peroneus longus and medial gastrocnemius muscles showed higher activity during standing than sitting in both participants with and without neck pain. Within the neck pain group, tibialis anterior activation was significantly higher during standing than during sitting and writing. Exploratory correlations between UT and ankle muscles showed different patterns by group and task but did not establish altered neuromuscular control. Larger studies are needed to confirm these findings and determine their clinical relevance.

## Figures and Tables

**Table 1:** Median values of the demographic characteristics of the two study groups.

Variables	GA Group (*n* = 8), Median	GB Group B (*n* = 19), Median
**Age (years)**	19	19
**Weight (kg)**	56	53
**Height (cm)**	168	165
**BMI (kg/h^2^)**	19.84	19.47
**CVA from sitting position**	54	50.5
**CVA from standing position**	50	55

kg: kilogram, cm: centimeter, h: height, *n*: number of students, CVA: craniovertebral angle.

**Table 2:** Mean rank values of demographic variables for both study groups.

Variables	GA Group (*n* = 8)	GB Group (*n* = 19)	*p* Value
	Mean Rank	Mean Rank	
**Age (years)**	10.65	16.64	0.053
**Weight (kg)**	15.25	11.5	0.150
**Height (cm)**	16	11	0.082
**BMI (kg/h^2^)**	14.5	12.00	0.385
**CVA from sitting position**	6	5.17	0.669
**CVA from standing position**	3.88	6.58	0.163

kg: kilogram, cm: centimeter, h: height, *n*: number of students, CVA: craniovertebral angle.

**Table 3:** UT statistical analysis between groups.

(I) Group	(J) Group	Mean Difference (I–J)	Std. Error	Sig.	95% Confidence Interval for Difference	95% Confidence Interval for Difference
					Lower Bound	Upper Bound
GB	GA	6.117 *	1.549	<0.001 *	3.045	9.190
Right	Left	1.391	1.549	0.371	−1.682	4.463
Sitting	Standing	4.501	1.897	0.059	−0.116	9.119
	Writing	−1.028	1.897	1.000	−5.646	3.590
Standing	Sitting	−4.501	1.897	0.059	−9.119	0.116
	Writing	−5.530 *	1.897	0.013 *	−10.148	−0.912

* = Significant.

**Table 4:** UT statistical analysis within GB.

(I) Condition	(J) Condition	Mean Difference (I–J)	Std. Error	Sig.	95% Confidence Interval for Difference	95% Confidence Interval
					Lower Bound	Upper Bound
Sitting	Standing	5.937 *	1.845	0.006 *	1.430	10.443
	Writing	−3.276	1.845	0.238	−7.782	1.231
Standing	Sitting	−5.937 *	1.845	0.006 *	−10.443	−1.430
	Writing	−9.212 *	1.845	<0.001 *	−13.719	−4.706
Right	Left	4.932 *	1.506	0.002 *	1.937	7.928

* = Significant.

**Table 5:** PL statistical analysis between groups.

(I) Condition	(J) Condition	Mean Difference (I–J)	Std. Error	Sig.	95% Confidence Interval for Difference	95% Confidence Interval for Difference
					Lower Bound	Upper Bound
**Sitting**	Standing	−6.541 *	1.973	0.004 *	−11.343	−1.739
	Writing	−2.962	1.973	0.409	−7.764	1.840
**Standing**	Sitting	6.541 *	1.973	0.004 *	1.739	11.343
	Writing	3.579	1.973	0.218	−1.223	8.381

* = Significant.

**Table 6:** PL statistical analysis within groups.

(I) Condition	(J) Condition	Mean Difference (I–J)	Std. Error	Sig.	95% Confidence Interval for Difference	95% Confidence Interval for Difference
					Lower Bound	Upper Bound
**Sitting GB**	Standing	−5.655 *	1.948	0.014 *	−10.414	−0.895
	Writing	−4.830 *	1.948	0.045 *	−9.590	−0.071
**Standing GB**	Sitting	5.655 *	1.948	0.014 *	0.895	10.414
	Writing	0.824	1.948	1.000	−3.935	5.584
**Sitting GA**	Standing	−7.427 *	1.792	0.002 *	−12.158	−2.697
	Writing	−1.094	1.792	1.000	−5.824	3.637
**Standing GA**	Sitting	7.427 *	1.792	0.002 *	2.697	12.158
	Writing	6.334 *	1.792	0.007 *	1.603	11.064

* = Significant.

**Table 7:** MGC statistical analysis between groups.

(I) Condition	(J) Condition	Mean Difference (I–J)	Std. Error	Sig.	95% Confidence Interval for Difference	95% Confidence Interval for Difference
					Lower Bound	Upper Bound
**Sitting**	Standing	−3.392 *	0.866	<0.001 *	−5.501	−1.282
	Writing	−1.021	0.866	0.723	−3.131	1.088
**Standing**	Sitting	3.392 *	0.866	<0.001 *	1.282	5.501
	Writing	2.370 *	0.866	0.022 *	0.260	4.480

* = Significant.

**Table 8:** MGC statistical analysis within groups.

(I) Condition	(J) Condition	Mean Difference (I–J)	Std. Error	Sig.	95% Confidence Interval for Difference	95% Confidence Interval for Difference
					Lower Bound	Upper Bound
**Sitting GB**	Standing	−3.181 *	0.746	<0.001 *	−5.004	−1.358
	Writing	−2.013 *	0.746	0.025 *	−3.836	−0.190
**Standing GB**	Sitting	3.181 *	0.746	<0.001 *	1.358	5.004
	Writing	1.168	0.746	0.364	−0.655	2.991
**Sitting GA**	Standing	−3.602 *	0.710	<0.001 *	−5.576	−1.628
	Writing	−0.030	0.710	1.000	−2.004	1.944
**Standing GA**	Sitting	3.602 *	0.710	<0.001 *	1.628	5.576
	Writing	3.572 *	0.710	<0.001 *	1.598	5.546

* = Significant.

**Table 9:** TA statistical analysis within GB.

(I) Condition	(J) Condition	Mean Difference (I–J)	Std. Error	Sig.	95% Confidence Interval for Difference Lower Bound	95% Confidence Interval for Difference Upper Bound
**Sitting**	Standing	−2.905 *	0.809	0.002 *	−4.882	−0.929
	Writing	−0.818	0.809	0.945	−2.795	1.159
**Standing**	Sitting	2.905 *	0.809	0.002	0.929	4.882
	Writing	2.087 *	0.809	0.035 *	0.111	4.064

* = Significant.

**Table 10:** Correlations between UT and ankle muscle mean amplitude sEMG_RMS_ values from different positions.

	Muscles	Spearman Correlation	Significant
**UT Sitting (GB)**	PL	0.522	0.003 *
	MGC	0.406	0.026 *
	TA	0.397	0.030 *
**UT Standing (GB)**	PL	0.587	<0.001 *
	MGC	0.128	0.501
	TA	0.548	0.002 *
**UT Writing (GB)**	PL	0.396	0.030 *
	MGC	0.512	0.004 *
	TA	0.200	0.290
**UT Sitting (GA)**	PL	−0.341	0.408
	MGC	−0.394	0.440
	TA	−0.366	0.373
**UT Standing (GA)**	PL	0.610	0.108
	MGC	−0.939	0.005 *
	TA	0.610	0.108
**UT Writing (GA)**	PL	−10.00	0.000 *
	MGC	−0.939	0.005 *
	TA	−0.805	0.016 *

* = Significant.

**Table 11:** Correlations between CVA and mean amplitude sEMG_RMS_ values.

	Muscles	Spearman Correlation	Significant
**CVA from Standing (GB)**	UT	0.696	0.125
	PL	−0.812	0.050 *
	MGC	−0.522	0.288
	TA	0.058	0.913
**CVA from Standing (GA)**	UT	0.775	0.225
	PL	0.738	0.262
	MGC	−0.866	0.333
	TA	0.738	0.262
**CVA from Sitting (GB)**	UT	−0.029	0.957
	PL	0.314	0.544
	MGC	0.200	0.704
	TA	−0.029	0.957
**CVA from Sitting (GA)**	UT	0.889	0.111
	PL	0.000	1.000
	MGC	0.866	0.333
	TA	0.000	1.000

* = Significant.

## Data Availability

Data are unavailable due to privacy or ethical restrictions; however, data can be provided by the corresponding author under reasonable request.

## Abbreviations

The following abbreviations are used in this manuscript:

UT	Upper Trapezius
MGC	Medial Gastrocnemius
PL	Peroneus Longus
TA	Tibialis Anterior
GB	Neck Pain group
GA	Control Group
NRS	Numerical Rating Scale
NDI	Neck Disability Index
sEMG	Surface Electromyography
NP	Neck Pain
FHP	Forward Head Posture
sEMGRMS	Surface Electromyography Root Mean Square
CVA	Craniovertebral Angle
MTrPs	Myofascial Trigger Point
